# Tissue-type plasminogen activator contributes to remodeling of the rat ductus arteriosus

**DOI:** 10.1371/journal.pone.0190871

**Published:** 2018-01-05

**Authors:** Junichi Saito, Utako Yokoyama, Naoki Nicho, Yun-Wen Zheng, Yasuhiro Ichikawa, Satoko Ito, Masanari Umemura, Takayuki Fujita, Shuichi Ito, Hideki Taniguchi, Toshihide Asou, Munetaka Masuda, Yoshihiro Ishikawa

**Affiliations:** 1 Cardiovascular Research Institute, Yokohama City University, Yokohama, Japan; 2 Department of Regenerative Medicine, Yokohama City University, Yokohama, Japan; 3 Department of Pediatrics, Graduate School of Medicine, Yokohama City University, Yokohama, Japan; 4 Department of Cardiovascular Surgery, Kanagawa Children’s Medical Center, Yokohama, Japan; 5 Department of Surgery, Yokohama City University, Yokohama, Japan; Osaka Shiritsu Daigaku, JAPAN

## Abstract

**Aims:**

The ductus arteriosus (DA) closes after birth to adapt to the robust changes in hemodynamics, which require intimal thickening (IT) to occur. The smooth muscle cells of the DA have been reported to play important roles in IT formation. However, the roles of the endothelial cells (ECs) have not been fully investigated. We herein focused on tissue-type plasminogen activator (t-PA), which is a DA EC dominant gene, and investigated its contribution to IT formation in the DA.

**Methods and results:**

ECs from the DA and aorta were isolated from fetal rats using fluorescence-activated cell sorting. RT-PCR showed that the t-PA mRNA expression level was 2.7-fold higher in DA ECs than in aortic ECs from full-term rat fetuses (gestational day 21). A strong immunoreaction for t-PA was detected in pre-term and full-term rat DA ECs. t-PA-mediated plasminogen-plasmin conversion activates gelatinase matrix metalloproteinases (MMPs). Gelatin zymography revealed that plasminogen supplementation significantly promoted activation of the elastolytic enzyme MMP-2 in rat DA ECs. *In situ* zymography demonstrated that marked gelatinase activity was observed at the site of disruption in the internal elastic laminae (IEL) in full-term rat DA. In a three-dimensional vascular model, EC-mediated plasminogen-plasmin conversion augmented the IEL disruption. *In vivo* administration of plasminogen to pre-term rat fetuses (gestational day 19), in which IT is poorly formed, promoted IEL disruption accompanied by gelatinase activation and enhanced IT formation in the DA. Additionally, experiments using five human DA tissues demonstrated that the t-PA expression level was 3.7-fold higher in the IT area than in the tunica media. t-PA protein expression and gelatinase activity were also detected in the IT area of the human DAs.

**Conclusion:**

t-PA expressed in ECs may help to form IT of the DA via activation of MMP-2 and disruption of IEL.

## Introduction

The ductus arteriosus (DA) is a fetal arterial bypass between the pulmonary artery and the descending aorta, and it is essential for maintaining fetal life. In full-term infants, the DA normally closes during the first few days after birth. In pre-term infants, however, the DA often remains patent after the neonatal period. Persistent patent DA (PDA) occurs in up to 65% of extremely low birth weight infants [1], and is associated with systemic hypo-perfusion, pulmonary congestion, and a high mortality rate [2, 3]. Although more than 50% of extremely preterm infants receive pharmacological therapy with cyclooxygenase inhibitors or surgical ligation of the DA, the current strategies for PDA still require improvement [4]. Thus, supportive therapies to improve the prognosis of PDA are needed.

In addition to DA smooth muscle contraction, we and others suggested that ductal tissue remodeling, such as intimal thickening (IT) formation, is necessary for complete anatomical closure of the DA [5–17]. In humans, IT gradually developed mid-gestation and prominent IT is observed in the full-term DA. IT formation is not fully developed in preterm infants [17–19], and it is attenuated in full term infants with PDA [15]. The DA undergoes several sequential processes of IT formation toward birth [7]. Disruption of the internal elastic lamina (IEL) and subendothelial edema are the early processes of DA remodeling [7]. In the human DA, IEL disruption starts at around 17 weeks gestation and becomes increasingly evident between 22 and 26 weeks gestation, when vascular smooth muscle cells (SMCs) migrate into the subendothelial region through the disrupted IEL. IT becomes more prominent at the site of the disrupted IEL [18–20]. The IEL is then frequently disrupted between 27 and 34 weeks gestation [7, 17], and further SMC migration promotes DA luminal narrowing during the late gestational period. These histological findings in human DAs suggest that extremely preterm infants born at less than 27 weeks gestation, who frequently have PDA, have incomplete disruption of the DA IEL. We previously reported that prostaglandin E receptor EP4 inhibited elastogenesis in the tunica media of the DA [8, 21], but the molecular mechanisms of IEL disruption remain largely unknown.

Although SMCs play important roles in the process of IT formation [7, 9, 10, 17], the roles of the endothelial cells (ECs), which are adjacent to the IEL, have not been fully investigated. Recently, we reported the transcriptional profiles of the rat DA ECs and revealed the DA EC dominant genes [22]. We herein focused on tissue-type plasminogen activator (t-PA) and investigated its role in IEL and IT formation.

## Materials and methods

### Animal studies

Wistar rat fetuses were obtained from timed-pregnant rats that were purchased from Japan SLC Inc. (Shizuoka, Japan). All animal studies were approved by the Institutional Animal Care and Use Committees of Yokohama City University in accordance with the Guide for the Care and Use of Laboratory Animals (reference number: F-A-16-010).

### Human studies

The protocol for using human DA tissues was approved by the human subject committees at Yokohama City University and Kanagawa Children’s Medical Center (reference number: B150305001 and 1502–05, respectively), and conformed to the principles outlined in the Declaration of Helsinki. Human DA tissues were obtained from patients with congenital heart diseases during cardiac surgery in Yokohama City University or Kanagawa Children’s Medical Center. All samples were obtained after receiving the parent’s or guardian’s written informed consent. The patient information is summarized in S1 Table.

### Fluorescence-activated cell sorting

Endothelial cells (ECs) were obtained by fluorescence-activated cell sorting (FACS) as described previously [11, 22]. Briefly, pooled tissues of the DA and aorta from approximately 30 Wister rat fetuses (gestational day 21) were subjected to FACS to obtain one sample. DA and aorta tissues were isolated from the same fetal rats. We repeated FACS and obtained six samples (total of about 180 fetuses) for quantitative reverse transcription-polymerase chain reaction (RT-PCR) and four samples (total of about 120 fetuses) for gelatin zymography.

After cell dispersion by collagenase-dispase enzyme mixture, cell suspensions were subjected to FACS analysis using FITC-conjugated anti-CD31 antibodies (Abcam, Cambridge, MA, USA) and APC/Cy7-conjugated anti-CD45 antibodies (BioLegend, San Diego, CA, USA) as cell surface markers for ECs and hematopoietic derivation cells, respectively. The cells in anti-CD31-positive and anti-CD45-negative (CD31^+^/CD45^−^) areas were defined as ECs (S1 Fig). All cells were detected and sorted using BD FACS Aria™II (Becton Dickinson, San Jose, CA, USA).

### RNA isolation and quantitative reverse transcription-polymerase chain reaction

Total RNA was isolated from the cells sorted by FACS using an RNAiso plus kit (Takara, Tokyo, Japan), according to the manufacturer’s instructions. RT-PCR was performed as described previously [6]. The primers were designed between multiple exons based on the rat and human nucleotide sequences of t-PA (5′-CTCAGACAACTTACCAACAGCATC-3′ and 5′-ATCACAGCGTTTCCCAACA-3′ for rat t-PA, and 5′-CGCAGATGATATACCAGCAACA-3′ and 5′-ACAGCACTTCCCAGCAAATC-3′ for human t-PA). The abundance of each gene was determined relative to 18S rRNA.

### RNA interference

Small interfering RNA (siRNA) of human t-PA and control siRNA were purchased from Thermo Fisher Scientific (Waltham, MA, USA). According to the manufacturer’s instructions, cultured human umbilical vein ECs (HUVECs) were transfected with siRNA targeted for t-PA using Lipofectamine RNAiMAX (Invitrogen, Waltham, MA, USA). We examined t-PA mRNA expression using quantitative RT-PCR.

### Tissue staining and immunohistochemistry

To determine IEL and IT formation, paraffin tissue sections were stained with elastica van Gieson, as recommended by the manufacturer (Muto Pure Chemicals, Tokyo, Japan). Immunohistochemical analysis was performed as described previously [8, 9]. Rabbit polyclonal anti-t-PA (Santa Cruz Biotechnology, Dallas, TX, USA), rabbit polyclonal anti-von Willebrand factor (Dako Cytomation, Santa Clara, CA, USA), mouse monoclonal anti-α-SMA (Sigma-Aldrich, St. Louis, MO, USA), rabbit polyclonal anti-MMP2 (Novus Biologicals, Little, CO, USA) antibodies were used.

### Scoring of IEL disruption in rat DA

We defined the degree of IEL disruption in the rat DA as follows: Grade 0 (score 0), IEL are continuously aligned between ECs and SMCs; Grade 1 (score 1), partial disruption of IEL is observed; and Grade 2 (score 2), multiple disruptions of IEL are observed. Each DA tissue was evenly divided into eight parts, and an individual score was determined for all eight parts in a blinded manner (S3 Fig). The total score of each DA was the sum of the scores for each of the parts (maximum score, 16). We used 6–8 rats per group for the analysis. The average score of three sections per rat were used.

### Gelatin zymography

As described above, rat ECs from both the DA and the aorta were obtained using FACS. These ECs were adjusted to 7.0 × 10^3^ cells/100 μl of medium and seeded in a 96-well plate. We cultured the ECs in the presence or absence of plasminogen (10 μg/ml) (WAKO, Osaka, Japan) for 72 h. The conditioned medium was subjected to gelatin zymography, as described previously [23].

### *In situ* gelatin zymography

Fetal arteries, including the DA and the aorta, were removed from the thoracic cavity. Fetal arterial tissues and three-dimensional (3D) vascular multilayers were fixed in a zinc-based fixative for 36 h and embedded in paraffin [24]. The paraffin-embedded tissue sections (thickness, 8 μm) were heated at 59°C overnight, deparaffinized in xylene, and rehydrated in graded alcohol baths. Dissolved DQ gelatin (20 μg/ml) (Invitrogen, Waltham, MA, USA) was added on the tissue sections, and they were then incubated for 2 h in a dark, humid chamber. The sections were then rinsed with double-distilled water and fixed in 4% neutral-buffered formalin for 10 min in the dark. Sections were then rinsed twice in phosphate-buffered saline (PBS), and DAPI (4′, 6-diamidino-2-phenylindole) (Invitrogen, Waltham, MA, USA) was added to stain the nuclei. After 20 min, sections were rinsed in PBS and mounted using aqueous mounting medium. To verify the contribution of matrix metalloprotease (MMP), control slides were pre-incubated with ethylenediaminetetraacetic acid (EDTA, 20 mM) for 1 h. EDTA (20 mM) was also added to the substrate. Fluorescence was detected using a FV1000 confocal laser microscope (Olympus, Tokyo, Japan).

### Construction of a 3D vascular model

To examine disruption of elastic laminae by conversion of plasminogen to plasmin, we constructed a 3D vascular model, as described previously [25, 26]. Seven-layered 3D multilayers of neonatal rat SMCs were constructed for one week, and incubated in 1% fetal bovine serum (FBS)/Dulbecco’s modified Eagle’s medium (DMEM, Sigma-Aldrich, St. Louis, MO, USA) for 48 h. HUVECs (JCRB Cell Bank, Osaka, Japan) were then seeded on top of the multilayers at a density of 14 × 10^4^ cells/cm^2^. Culture medium was changed to a mixture of DMEM and an equal amount of endothelial cell growth medium (PromoCell, Heidelberg, Germany) containing 1% FBS and cultured for 48 h. The 3D multilayers were then incubated in the presence or absence of plasminogen (10 μg/ml). Forty-eight hours after incubation, we fixed the 3D multilayers in buffered 10% formalin or zinc-based fixative and embedded them in paraffin.

### *In vivo* administration of plasminogen and IT formation analysis

After pregnant rats were anesthetized with isoflurane, *in utero* fetuses on gestational day 19 were injected intraperitoneally with PBS (100 μl) or plasminogen (20 μg/100 μl PBS). Twenty-four hours after plasminogen administration, the pregnant rats were euthanized by injection of pentobarbital (200 mg/kg). Immediately after euthanasia, the fetuses were obtained (gestational day 20). After decapitation, fetal arteries, including the DA and the aortic arch, were removed from the thoracic cavity and fixed in buffered 10% formalin or zinc-based fixative and embedded in paraffin. The sectioned segments in the middle portion of the DA were used for *in situ* zymography and elastica van Gieson staining. IT formation was quantified using Image J software, as described previously [9]. Briefly, IT was defined as (neointima area / media area) × 100%. The average of at least three sections was used for the value for each tissue.

### Statistical analysis

All values are shown as the mean ± standard error of the mean (SEM) of more than three independent experiments. Statistical analysis was performed between two groups using an unpaired *t*-test with Welch’s correction. A value of *p* < 0.05 was considered significant.

## Results

### Tissue-type plasminogen activator was highly expressed in ECs of the rat DA

**Fig 1:**
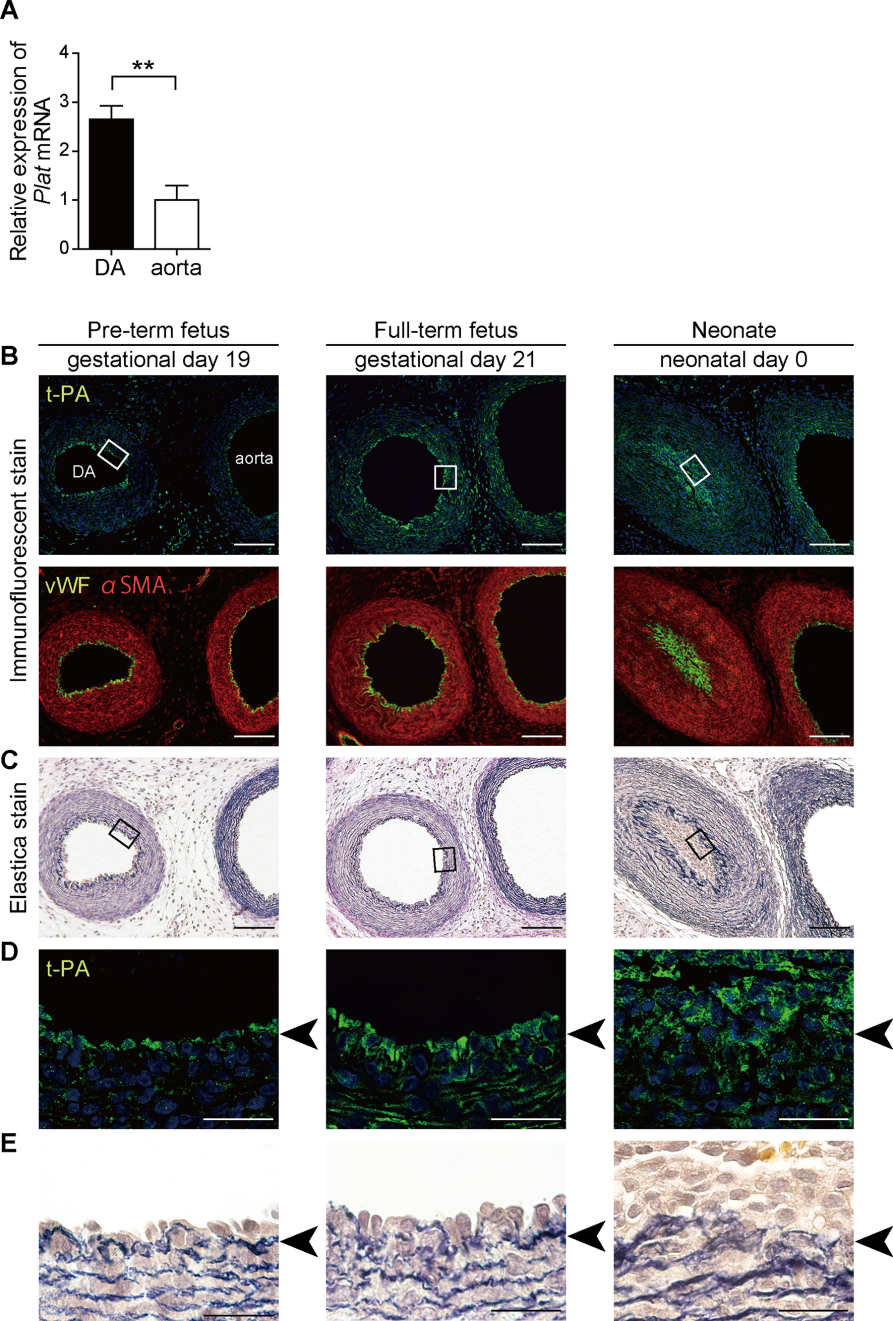
t-PA expression in ECs of the rat DA and aorta. **(A)** Expression of *Plat* mRNA detected using quantitative RT-PCR. n = 6, **p < 0.01. **(B)** Immunofluorescence for t-PA protein (green color) in the rat DA on gestational days 19 and 21 and neonatal day 0 are shown in the upper panels. Blue color indicates nuclei. Co-immunofluorescence staining for von Willebrand factor (vWF, green color) and α-smooth muscle actin (αSMA, red) are shown in the lower panels. **(C)** Elastica van Gieson staining images for visualization of elastic fibers. Boxes indicate the areas of the lower panels. Scale bars, 100 μm. **(D-E)** Higher magnification of the upper panels from B and C is shown. Arrows indicate IEL. Scale bars, 20 μm.

DA ECs and aortic ECs were isolated from full-term fetal rats (gestational day 21) using FACS. Quantitative RT-PCR showed t-PA (*Plat*) mRNA expression was significantly higher in DA ECs than in aortic ECs (Fig 1A), which is in accordance with our previous report using microarray analysis [27]. Immunofluorescence of the rat tissues on gestational days 19 and 21 and neonatal day 0 showed that t-PA proteins were abundantly expressed in ECs of the DA throughout this developmental period (Fig 1B and 1D). Elastic laminae were slightly disrupted in the DA on gestational day 19, and were fragmented on gestational day 21 (Fig 1C and 1E). t-PA protein expression was also confirmed in aortic ECs, but it seemed that t-PA expression was higher in the DA than in the aorta on gestational days 19 and 21, which was partially confirmed by the t-PA mRNA expression data shown in Fig 1A.

### Gelatinase activity in the rat DA

**Fig 2:**
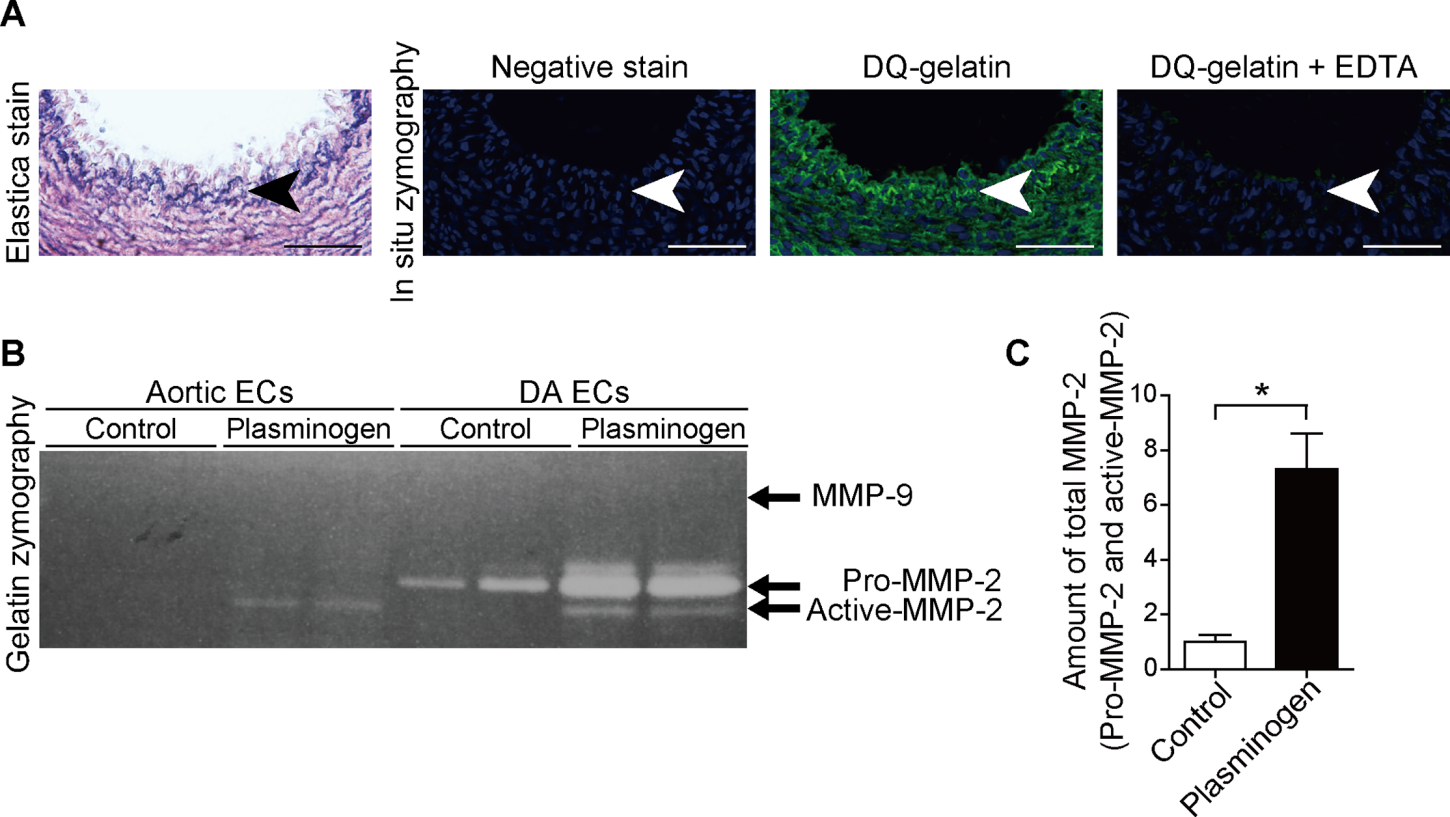
Gelatinase activity in the rat DA. **(A)** Elastica van Gieson staining of rat DA on gestational day 21 (left panel). *In situ* gelatin zymography using the serial sections demonstrates high gelatinase activity in IEL of the rat DA on gestational day 21. The green color indicates gelatinase activity (middle panel). Gelatinase activity was inhibited by EDTA supplementation (right panel). Arrows indicate IEL. The blue color of zymography indicates nuclei. Scale bars, 50 μm. **(B)** Gelatin zymography shows MMP activity in the conditioned medium cultured with ECs (gestational day 21). Active-MMP-2, pro-MMP-2, and MMP-9 are 62 kDa, 72 kDa, and 92 kDa, respectively. **(C)** Quantification of total MMP-2, including pro-MMP-2 and active-MMP-2, in DA ECs. (n = 6; *p < 0.05).

t-PA converts plasminogen into plasmin [28], which activates gelatinase MMP-2 [29] and MMP-9 [30]. It is well recognized that elastic laminae are degraded mainly by MMP-2 and MMP-9 [30]. We then investigated gelatinase activity in the rat DA tissue on gestational day 21. IEL was disrupted and gelatinase activity was marked in IEL of the DA, which was inhibited by the MMP inhibitor EDTA (Fig 2A).

Gelatin zymography demonstrated that the pro-MMP-2 level was higher in rat DA ECs than in aortic ECs under basal conditions (Fig 2B, Control). In the presence of plasminogen, greater MMP-2 activation was detected in DA ECs compared with aortic ECs (Fig 2B, Plasminogen). Plasminogen supplementation significantly increased the amount of pro-MMP-2 and active MMP-2 in DA ECs (Fig 2C). MMP-9 was not detected in either DA ECs or aortic ECs (Fig 2B). These data indicate that the conversion of plasminogen to plasmin in DA ECs activates MMP-2.

### Elastic laminae were disrupted via MMP activation in the presence of plasminogen

**Fig 3:**
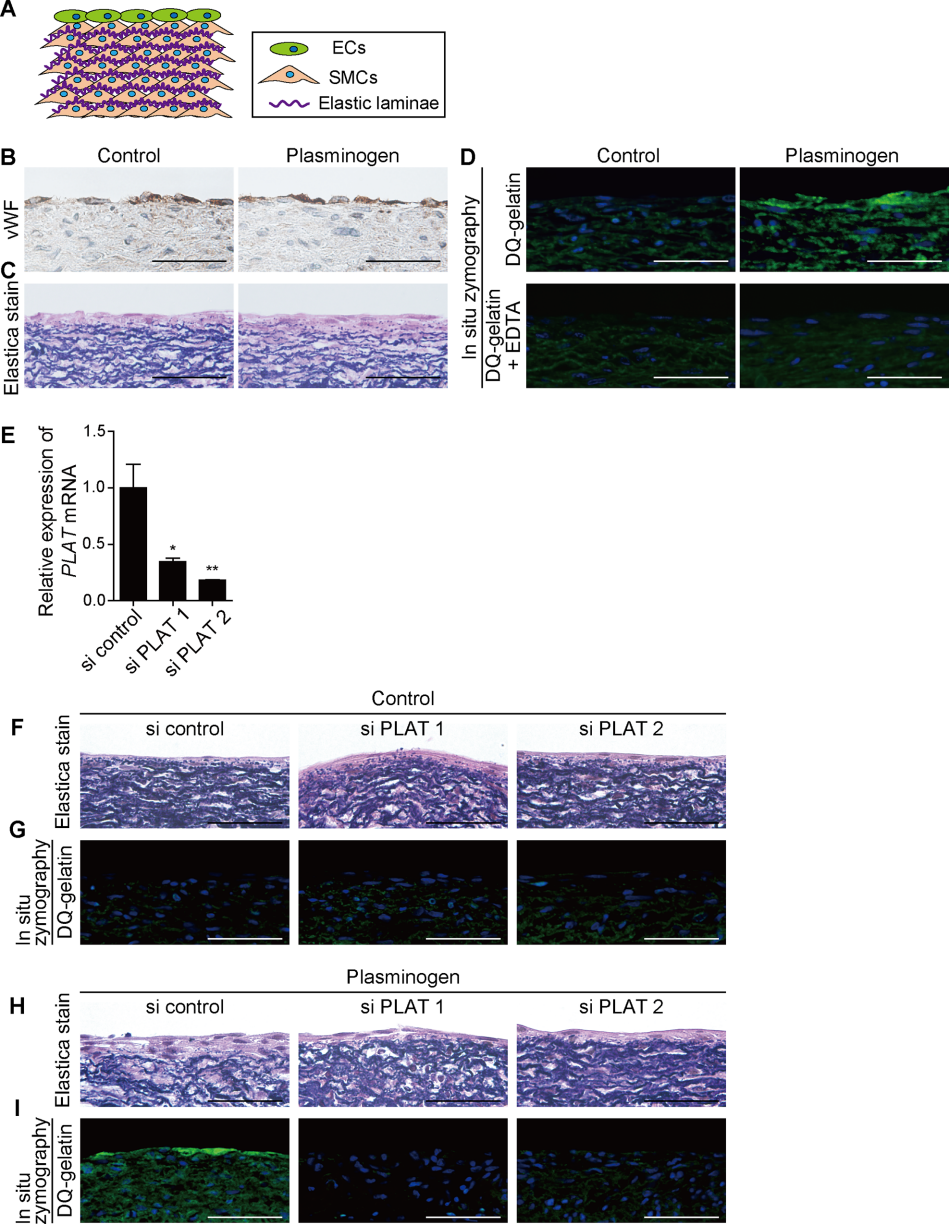
Elastic fibers were disrupted via MMP in the presence of plasminogen. **(A)** Scheme of a 3D vascular model containing HUVECs and rat neonatal aortic SMCs. **(B)** Immunohistochemistry shows the presence of ECs on multilayers of SMCs. The brown color indicates a positive immunoreaction for von Willebrand factor. Scale bars, 50 μm. **(C)** Elastica van Gieson staining shows layered elastic fibers indicated by a dark purple color. Scale bars, 50 μm. **(D)** *In situ* zymography of a plasminogen-treated 3D vascular model. The green color indicates gelatinase activity. The blue color indicates nuclei. Scale bars, 50 μm. **(E)** Quantitative RT-PCR shows a decrease in *PLAT* mRNA expression in HUVECs transfected with *PLAT*-targeted siRNA (si PLAT 1 or si PLAT 2). n = 4, *p < 0.05, **p < 0.01. **(F and H)** Elastica van Gieson staining shows that PLAT-targeted siRNAs attenuated plasminogen-induced disruption of elastic laminae. Scale bars, 50 μm. **(G and I)** *In situ* zymography shows that PLAT-targeted siRNAs attenuated plasminogen-induced gelatinase activity. Scale bars, 50 μm.

Although emerging evidence suggests that t-PA-mediated MMP activation promotes vascular remodeling [31], it is difficult to show t-PA-mediated disruption of elastic laminae *in vitro*. We examined the direct effect of plasminogen on elastic lamina disruption using a 3D vascular model that we developed [25, 26], in which ECs adhered on multilayers of SMCs containing layered elastic fibers (Fig 3A). In untreated 3D vascular models, von Willebrand factor-positive ECs were present on top of SMC multilayers (Fig 3B), and layered elastic laminae were observed underneath ECs (Fig 3C). Supplementation of plasminogen, a substrate of t-PA, however, promoted the disruption of elastic laminae and gelatinase activity, as demonstrated by elastica van Gieson staining and *in situ* zymography, respectively (Fig 3C and 3D). The enhanced gelatinase activity was attenuated by EDTA (Fig 3D).

To further examine the involvement of t-PA in plasminogen-induced disruption of elastic laminae, we used t-PA-targeted siRNAs. Two kinds of t-PA-targeted siRNAs significantly decreased *PLAT* mRNA expression in HUVECs (Fig 3E). Both t-PA-targeted siRNAs significantly attenuated plasminogen-induced disruption of elastic laminae and gelatinase activity in the 3D vascular models (Fig 3F and 3I).

ECs in the 3D vascular model were derived from HUVECs. Because HUVECs express abundant functional t-PA [32] and we confirmed MMP-2 activation in HUVECs by gelatin zymography (S2 Fig), these data support the concept that the conversion of plasminogen to plasmin promoted elastic lamina disruption via MMP activation.

### Plasminogen administration promoted IEL disruption and IT formation in the pre-term rat DA

We examined whether plasminogen administration promoted IEL disruption and IT formation in pre-term rat fetuses *in vivo*. The DA of pre-term rats (gestational day 19) had the same t-PA expression as that of full-term rats (gestational day 21 days) (1.00 ± 0.17 vs. 0.98 ± 0.31, n = 3, by quantitative RT-PCR), suggesting that the pre-term rat DA has the ability to convert plasminogen to plasmin.

**Fig 4:**
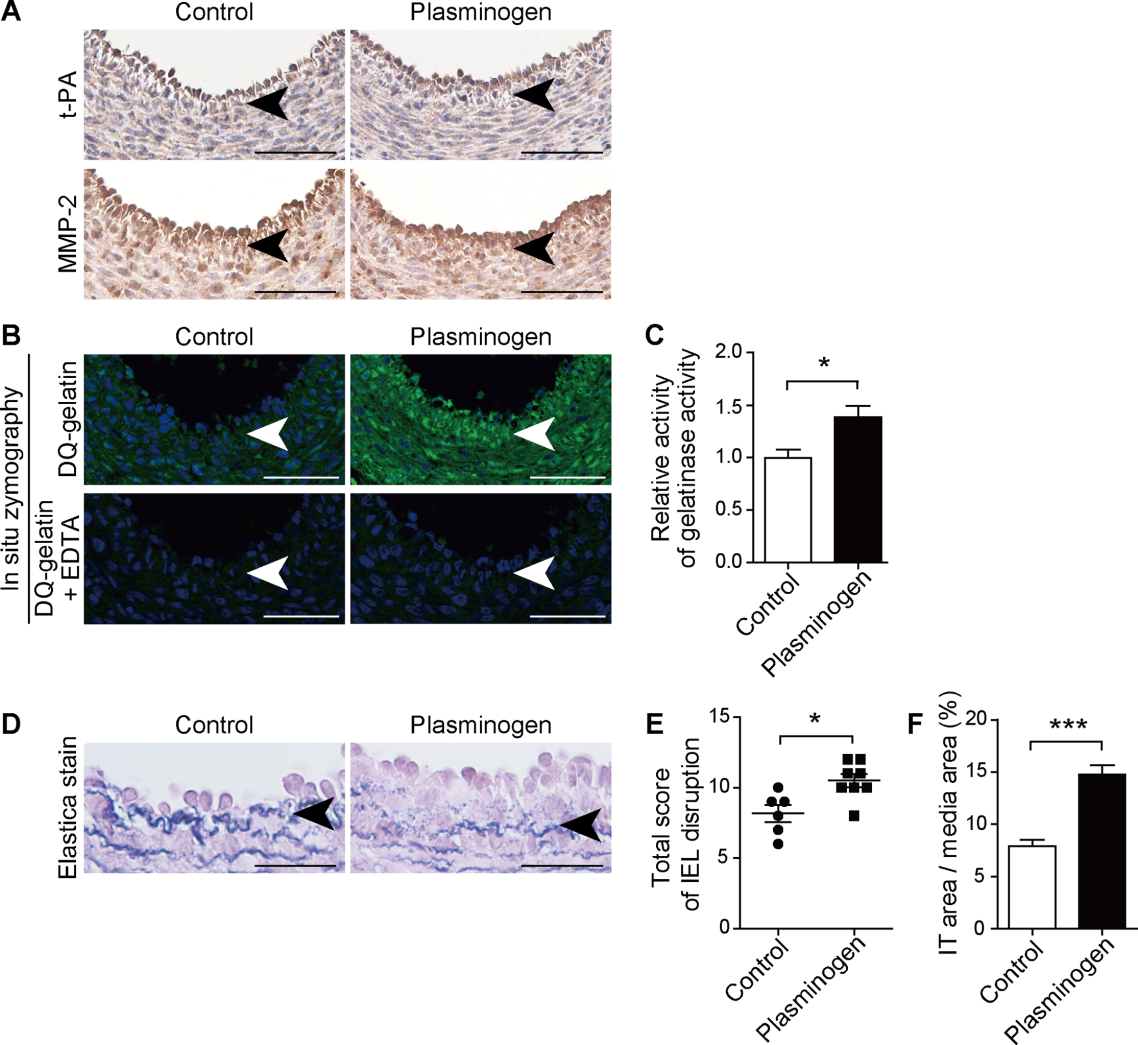
Plasminogen administration promoted IEL disruption, gelatinase activation and IT formation of the pre-term rat DA. **(A)** Immunohistochemistry shows the presence of t-PA and MMP-2 in the DA ECs. The brown color indicates a positive immunoreaction for t-PA and MMP-2. Scale bars, 100 μm. **(B)** *In situ* zymography demonstrates that plasminogen administration to fetal rats on gestational day 19 increased gelatinase activity, which was inhibited by EDTA. Scale bars, 100 μm. **(C)** Quantification of (B). n = 6, *p < 0.05. **(D)** Administration of plasminogen to fetal rats on gestational day 19 promoted IEL disruption and IT formation detected by elastica van Gieson staining. Arrows indicate IEL. Scale bars, 50 μm. **(E)** Plasminogen administration increased the total IEL disruption score in the rat DA. n = 6–8, *p < 0.05. **(F)** The IT area, normalized by the area of the tunica media, was increased by plasminogen administration. n = 6–8, ***p < 0.001.

We administered plasminogen to rat fetuses on gestational day 19, and analyzed them 24 h after administration. t-PA and MMP-2 protein expression were present in the area of ECs in PBS controls (Fig 4A). *In situ* zymography demonstrated that gelatinase activity was higher in the plasminogen-treated pre-term rat DA (Fig 4B and 4C). Enhanced gelatinase activity was attenuated by EDTA (Fig 4B), suggesting that conversion of plasminogen to plasmin activates MMP. In PBS controls, IEL disruption and IT formation was moderate and limited (Fig 4D). Conversely, plasminogen administration promoted elastic lamina disruption (Fig 4D), which was also confirmed by quantification using the scoring of IEL disruption (Fig 4E). IT formation was promoted in plasminogen-treated pre-term rat DA (Fig 4D and 4F). Representative scoring images of control or plasminogen-treated DAs are shown in S3 Fig.

### t-PA expression and gelatinase activation in IT of the human DA

**Fig 5:**
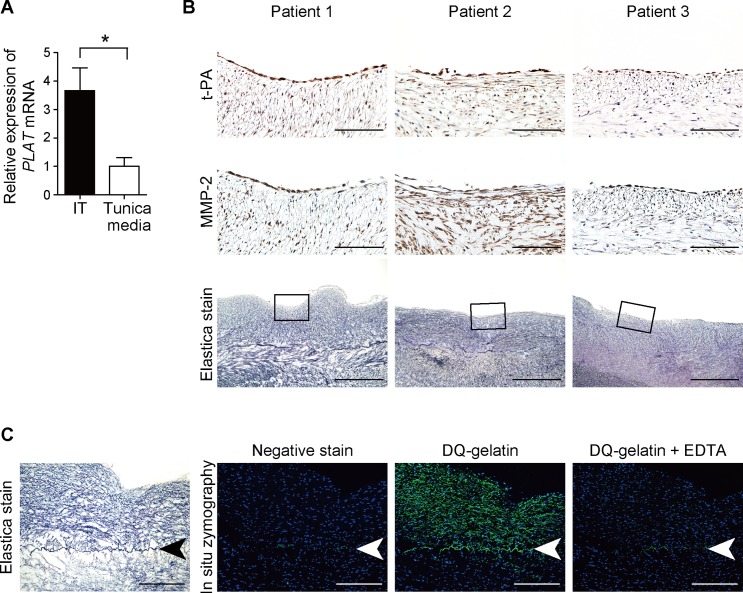
t-PA expression and gelatinase activity in IT of the human Das. **(A)** Quantitative RT-PCR shows higher *PLAT* mRNA expression in IT of the human DAs compared to the tunica media (Patients 1–5). n = 5, *p < 0.05. **(B)** Upper panels: Immunohistochemistry detected a positive immunoreaction for t-PA proteins in ECs and in the IT area in human DAs (Patients 1–3; brown color). Middle panels: Immunohistochemistry for MMP-2 in the human DAs. Lower panels: elastica van Gieson staining of the IT area in human DAs. Black boxes indicate the areas of the upper panels. Scale bars, 100 μm (upper panels) and 500 μm (lower panels). **(C)** *In situ* gelatin zymography demonstrates high gelatinase activity in the IEL and IT of the human DA (Patient 6). The green color indicates gelatinase activity (middle panel). Gelatinase activity was inhibited by EDTA supplementation (right panel). Profiles of Patients 1–6 are shown in S1 Table. Arrows indicate IEL. The blue color in zymography indicates nuclei. Scale bars, 200 μm.

We further examined t-PA expression in human DAs from five patients (Patients 1–5 in S1 Table) using quantitative RT-PCR. All patients needed a PGE_1_ infusion to keep the DA open and all the human DAs had prominent IT formation, suggesting that these DA samples were normally developed without delayed and/or dysregulated IT formation. The DA samples were manually divided into two parts: the inner part and the outer part. The inner part mainly contained IT, which consists of ECs and SMCs, and the outer part mainly contained the tunica media. We found higher t-PA expression in the inner part compared with the outer part (Fig 5A). Elastica van Gieson staining demonstrated prominent IT formation in the human DAs, and t-PA and MMP-2 proteins were highly expressed in IT of the human DAs (Patients 1–3 in S1 Table, Fig 5B). *In situ* zymography demonstrated enhanced gelatinase activity in the IT and the disrupted IEL, which was attenuated by EDTA (Patient 6 in S1 Table, Fig 5C). These findings indicate that t-PA may induce MMP activation in the area of human DA IT.

## Discussion

The present study suggests that t-PA is secreted from DA ECs and that it promotes the plasmin-induced activation of MMP-2 and the subsequent disruption of the IEL, which may contribute to IT formation in the DA. The results obtained from rat ECs further revealed that t-PA was dominantly expressed in the DA compared to the adjacent aorta. To the best of our knowledge, this is the first report of the functional roles of DA ECs in DA remodeling.

Our previous studies using microarray analyses of the rat DA and aorta demonstrated that t-PA was a DA dominant gene [22, 33]. t-PA mRNA expression was 2.4-fold higher in ECs isolated from full-term rat fetuses and neonates than in aortic ECs of fetuses at the same developmental stages [22]. In another study by our group using whole tissues of rat DA and the aorta, t-PA expression was detected in the rat DA on gestational day 19 and it was up-regulated gradually during development [33].

Levin et al. examined the developmental change in t-PA expression in Wistar rat fetuses and demonstrated that t-PA expression appeared in a transitory pattern that is restricted to arterial ECs [34]. Immunohistochemistry for t-PA using time-series fetal rats showed that t-PA expression in ECs started with the aorta and spread to large arteries on gestational day 13; by gestational day 15, t-PA expression was greatly decreased in ECs in the large arteries, including the aorta and main pulmonary artery [34]. The present study demonstrated abundant t-PA expression during mid-gestation and late-gestation in ECs of the rat DAs. t-PA was expressed in ECs of the aorta throughout the development examined in this study, but its expression seemed to be less than that in the DA after mid-gestation. Together with our previous studies, the present study suggests that t-PA is abundantly expressed in ECs of the DA compared to the aorta.

A recent study using the Mef2C-AHF-Cre; *ROSA*^*mTmG*^ strain suggested that ECs of aortic and pulmonary trunks and the DA were derived from the second heart field mesoderm [35]. Another line of study demonstrated that *Six2*-positive second heart field progenitors were identified in ECs of both aortic and pulmonary trunks, and that these progenitors were also present in the DA [36]. Our previous study demonstrated that ECs from the DA express genes associated with the second heart field [22]. These data suggest that ECs of the DA and arterial trunks shear the same cell lineage. However, our data demonstrated that the expression pattern of t-PA was not the same between the DA and the aorta, indicating that environmental factors, such as adjacent SMCs of the DA, may affect t-PA expression. Although changes in vessel wall mechanical stresses are known to promote t-PA expression [37], the t-PA spatial expression pattern cannot be explained solely by mechanical factors. The mechanisms of the dynamic changes of the t-PA spatio-temporal expression pattern during development remain to be studied.

IEL is a barrier between the intima and the tunica media, and its disruption is the early process of IT formation in the vascular system [38]. In the DA, IEL seems to be formed and thereafter progressively disrupted during fetal development [7]. Histological studies in the human DA demonstrated that, in the tunica media, elastogenesis is suppressed throughout the fetal period whereas a single continuous IEL was formed in the human DA before 17 weeks gestation [7, 39]. Disruption of IEL becomes prominent during 22 to 24 weeks gestation [7]. In addition to our previous study showing that prostaglandin E receptor EP4 signaling inhibited elastogenesis in the tunica media of the DA [8], the present study demonstrated a potential molecular mechanism of IEL disruption.

t-PA is a serine protease that converts the proenzyme plasminogen into plasmin [31]. Plasmin is also a serine protease that directly converts the following pro-MMPs into active-MMPs: MMP-1, MMP-3, MMP-9, and MMP-13 [30]. Although the exact role of plasmin in MMP-2 activation remains unclear, plasmin appears to cooperate with membrane-type 1 MMP (MT1-MMP) to fully activate pro-MMP-2 [29]. In the present study, MMP-2 activation was markedly enhanced in the presence of plasminogen in ECs of the DA, in which t-PA was highly expressed. These data suggest that t-PA in DA ECs promotes MMP-2 activation through plasminogen-plasmin conversion. In accordance with *in vitro* data, the *in vivo* administration of plasminogen to pre-term fetuses promoted gelatinase activity at the site of IEL and ECs. Because *in vivo* gelatinase activity was inhibited by EDTA, and *in vitro* data demonstrated MMP-2, but not MMP-9, activation, its activity is most likely attributable to MMP-2. Because MMP-2 degrades elastic fibers and collagen type VI, which are major components of IEL [30], MMP-2 appears to contribute to promotion of IEL disruption in the DA. Our previous study was unable to detect the enhanced activation of MMP-2 in whole cell lysate in rat DA [8]. This might be because MMP-2 activation was restricted in the IEL and IT of the DA.

The process of fetal DA remodeling occurs without inflammatory and coagulation processes, which is different from injury-induced IT formation in adult vessels. Therefore, to examine whether the presence of t-PA, but not inflammation and coagulation, in ECs promoted MMP activity and IEL degradation, we used a 3D vascular model consisting of HUVECs and rat neonatal aortic SMCs. HUVECs have high t-PA expression, and we confirmed that plasminogen administration increased MMP-2, but not MMP-9, activation in HUVECs. In this *in vitro* system, plasminogen supplementation promoted gelatinase activation and IEL degradation. These data suggest that the t-PA-mediated conversion of plasminogen to plasmin promoted IEL disruption, at least in an inflammation-free condition.

It is known that ECs produce tropoelastin and contribute to IEL formation together with SMCs [40]. Ideally, arterial ECs and SMCs should be used to study the regulation of IEL. However, it is not feasible to use arterial ECs because the 3D vascular model requires a substantial number of cells. We therefore used HUVECs, which are widely used as a research tool for ECs. Although tropoelastin expression was very low in HUVECs, we reported that rat neonatal aortic SMCs, which can be easily obtained, have the ability to form layered elastic laminae *in vitro* [25]. In the 3D vascular model, therefore, we suggest that the elastic laminae including the IEL were derived from SMCs. Although the 3D vascular model is an artificial *in vitro* system from the point of view of IEL formation, the present study indicates that this vascular model, which is composed of widely available cells, can be used to study interactions of ECs and elastic lamina formation.

Both hyaluronic acid accumulation in subendothelial region and IEL fragmentation are observed during early IT formation [20]. Our previous study of immunohistochemistry demonstrated that hyaluronic acid was not detected in ECs from fetal and neonatal rats [9]. Our preliminary data also showed that t-PA did not produce hyaluronic acid in HUVECs. Based on our studies and other published studies, t-PA does not seem to associate with hyaluronic acid production.

IT formation is necessary for complete closure of the DA. Strategies targeting IT formation have not yet been implemented, however. In the present study, plasminogen administration to fetal rats helped to form moderate IT of the DA *in vivo*. In human infants, serum plasminogen levels are only 50% of the adult values [41], and they are even lower in pre-term infants (30–36 weeks gestation) [42]. It was also reported that the enzymatic activity of fetal plasmin is lower than in the adult type of plasmin [43]. In the 1970s, plasminogen administration was used for very premature infants with respiratory distress syndrome because of its fibrinolytic effect. Ambrus et al. performed several double-blind, randomized clinical trials and found that plasminogen administration decreased severe respiratory distress and mortality without apparent side effects [44]. In this study, the effects of plasminogen on the DA were not assessed. Bhat et al. investigated the effect of plasminogen on surfactant-treated preterm lambs [45]. This study did not observe additional beneficial effects of plasminogen on respiratory distress, but no bleeding tendency was detected by intravenous plasminogen administration at a dose of 8 mg/kg body weight. We referred to this dose when deciding on the intraperitoneal dose of plasminogen in our study. Further study is required to investigate the role of t-PA in DA closure and the side effects of its systemic administration for clinical implications.

In conclusion, t-PA was highly expressed in ECs of rat and human DAs, and it is suggested that it promotes IEL disruption via MMP-2 activation, which may help to form IT of the DA.

## Supporting information

S1 Fig ECs were isolated from fetal tissues by FACS. **(A)** Flow cytometric analysis using FITC-conjugated anti-CD31 and APC/Cy7-conjugated anti-CD45 antibodies in the DA and the aorta of fetal rats. The gates R1 and R2 represent CD31^−^/CD45^−^ SMCs and CD31^+^/CD45^−^ ECs, respectively. Negative stain consists of the aortic cells without antibodies. **(B)** Total number of CD31^+^ cells in fetal rat tissues.
(TIF)

S2 Fig HUVECs had MMP-2 activity. Gelatin zymography shows MMP activity in the conditioned medium cultured with HUVECs. Active-MMP-2, pro-MMP-2, and MMP-9 are 62 kDa, 72 kDa, and 92 kDa, respectively.
(TIF)

S3 Fig Scoring of IEL disruption in rat DA. **(A)** Representative images of control or plasminogen-treated DAs *in vivo*. DA tissues were evenly divided into eight parts. The grade of IEL disruption (grade 0–2) is indicated in each part of the images. Black boxes indicate the areas of the lower panels. Scale bars, 100 μm. **(B)** Representative images of grade 0–2 (score 0–2). Scale bars, 20 μm.
(TIF)

S1 Table Summary of human patient profile. (DOCX)
