# Supplementary figures and images for: Tissue-type plasminogen activator contributes to remodeling of the rat ductus arteriosus
Source: PLoS One. 2018 Jan 5;13(1):e0190871. doi: 10.1371/journal.pone.0190871 (PMC5755942; doi:10.1371/journal.pone.0190871)

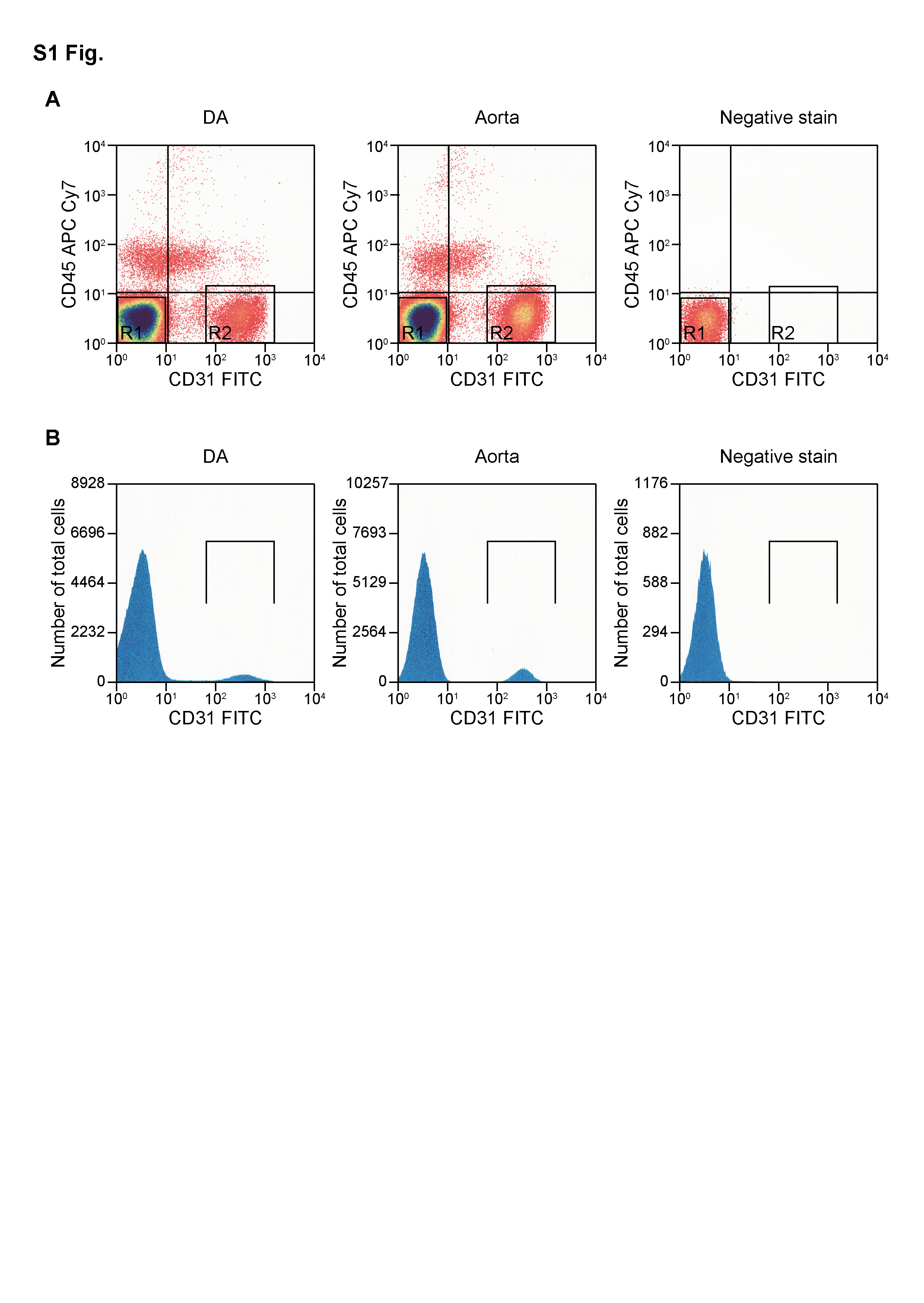

Supplement: S1 Fig — (A) Flow cytometric analysis using FITC-conjugated anti-CD31 and APC/Cy7-conjugated anti-CD45 antibodies in the DA and the aorta of fetal rats. The gates R1 and R2 represent CD31−/CD45− SMCs and CD31+/CD45− ECs, respectively. Negative stain consists of the aortic cells without antibodies. (B) Total number of CD31+ cells in fetal rat tissues. (TIF) [file pone.0190871.s001.tif]

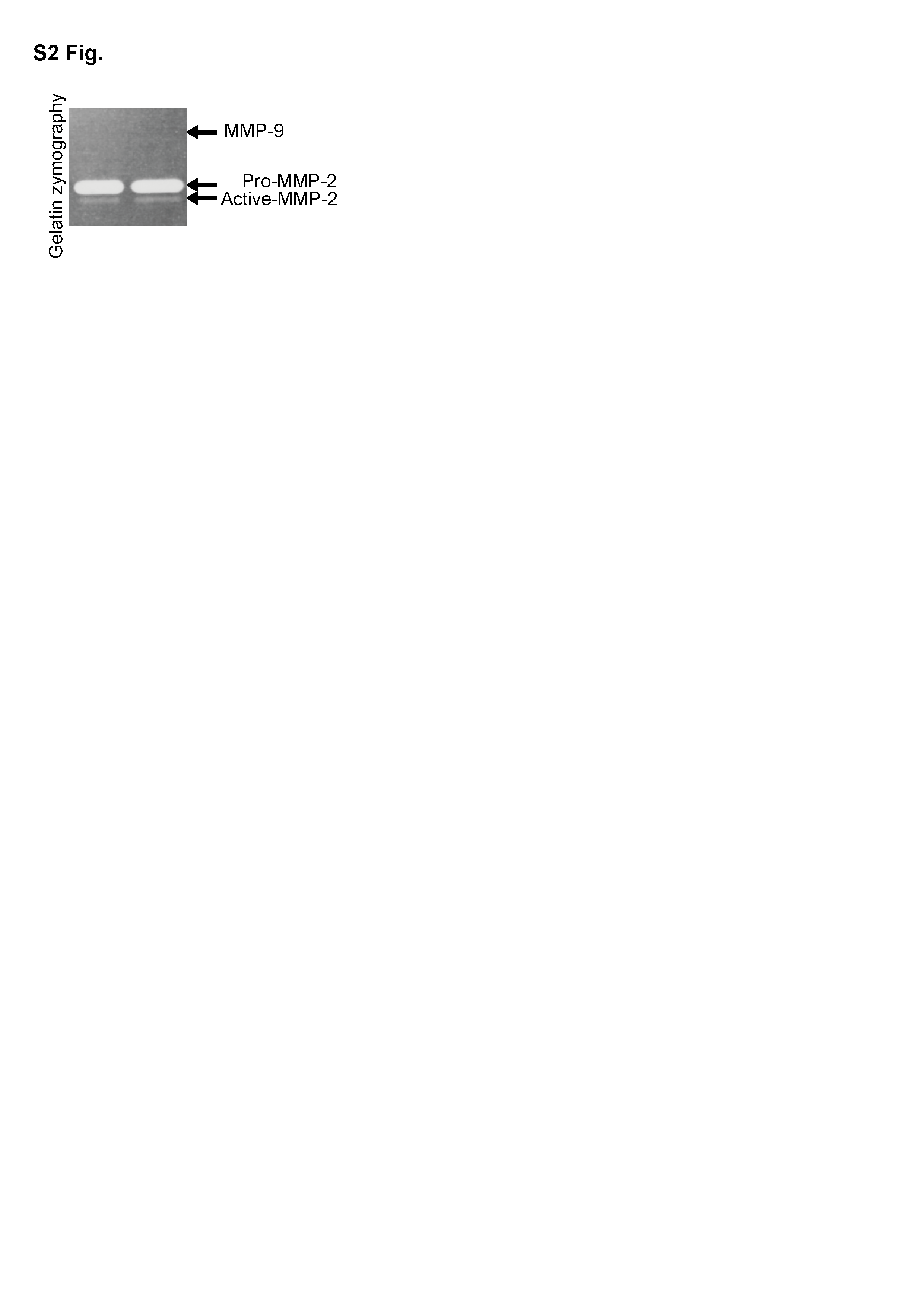

Supplement: S2 Fig — Gelatin zymography shows MMP activity in the conditioned medium cultured with HUVECs. Active-MMP-2, pro-MMP-2, and MMP-9 are 62 kDa, 72 kDa, and 92 kDa, respectively. (TIF) [file pone.0190871.s002.tif]

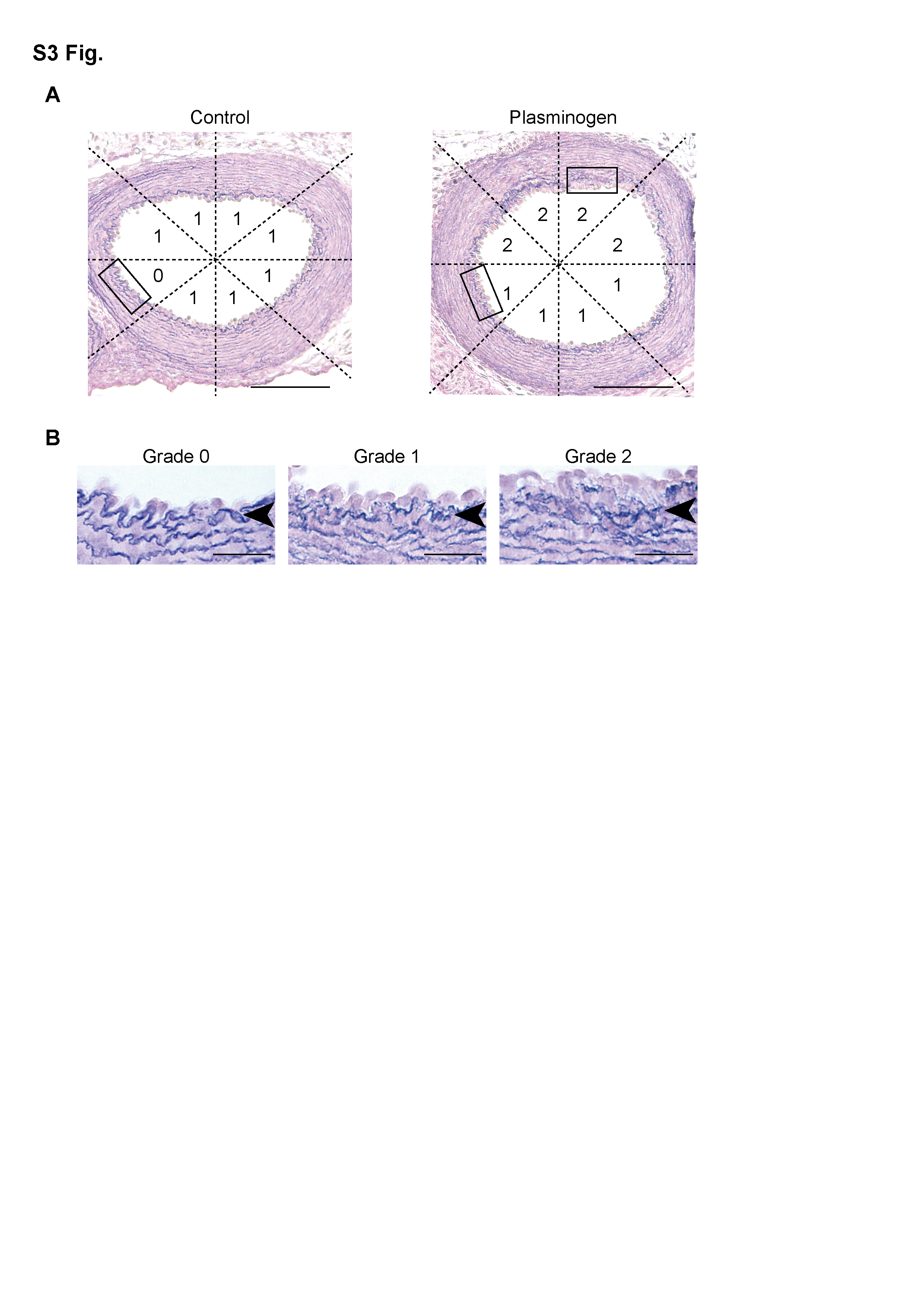

Supplement: S3 Fig — (A) Representative images of control or plasminogen-treated DAs in vivo. DA tissues were evenly divided into eight parts. The grade of IEL disruption (grade 0–2) is indicated in each part of the images. Black boxes indicate the areas of the lower panels. Scale bars, 100 μm. (B) Representative images of grade 0–2 (score 0–2). Scale bars, 20 μm. (TIF) [file pone.0190871.s003.tif]
