# Supplementary material for: Tissue-type plasminogen activator contributes to remodeling of the rat ductus arteriosus
Source: PLoS One. 2018 Jan 5;13(1):e0190871. doi: 10.1371/journal.pone.0190871 (PMC5755942; doi:10.1371/journal.pone.0190871)
Supplement: S1 Table — (DOCX) [file pone.0190871.s004.docx]

S1 Table.

| Patient | Diagnosis | Gestational Age  (weeks) | Birth Weight  (g) | Age at Operation  (days) | Preoperative Administration  of Prostaglandin E1 |
| --- | --- | --- | --- | --- | --- |
| 1 | Hypoplastic Left Heart Syndrome | 37 | 2654 | 97 | + |
| 2 | Hypoplastic Left Heart Syndrome | 40 | 3352 | 23 | + |
| 3 | Interruption of Aortic Arch, Ventricular Septal Defect | 39 | 2942 | 14 | + |
| 4 | Interruption of Aortic Arch, Ventricular Septal Defect | 39 | 2800 | 13 | + |
| 5 | Coarctation of the Aorta, Ventricular Septal Defect | 41 | 2948 | 4 | + |
| 6 | Interruption of Aortic Arch, Aortopulmonary Septal Defect | 38 | 3370 | 5 | + |
